# Supplementary figures and images for: Dietary Supplementation with Protocatechuic Acid and a Complex Eucommia ulmoides Leaf Extract Differentially Enhanced Reproductive Performance and Modulated Gut Microbiota in Late-Pregnancy Sows
Source: Animals (Basel). 2025 Oct 31;15(21):3166. doi: 10.3390/ani15213166 (PMC12610218; doi:10.3390/ani15213166)

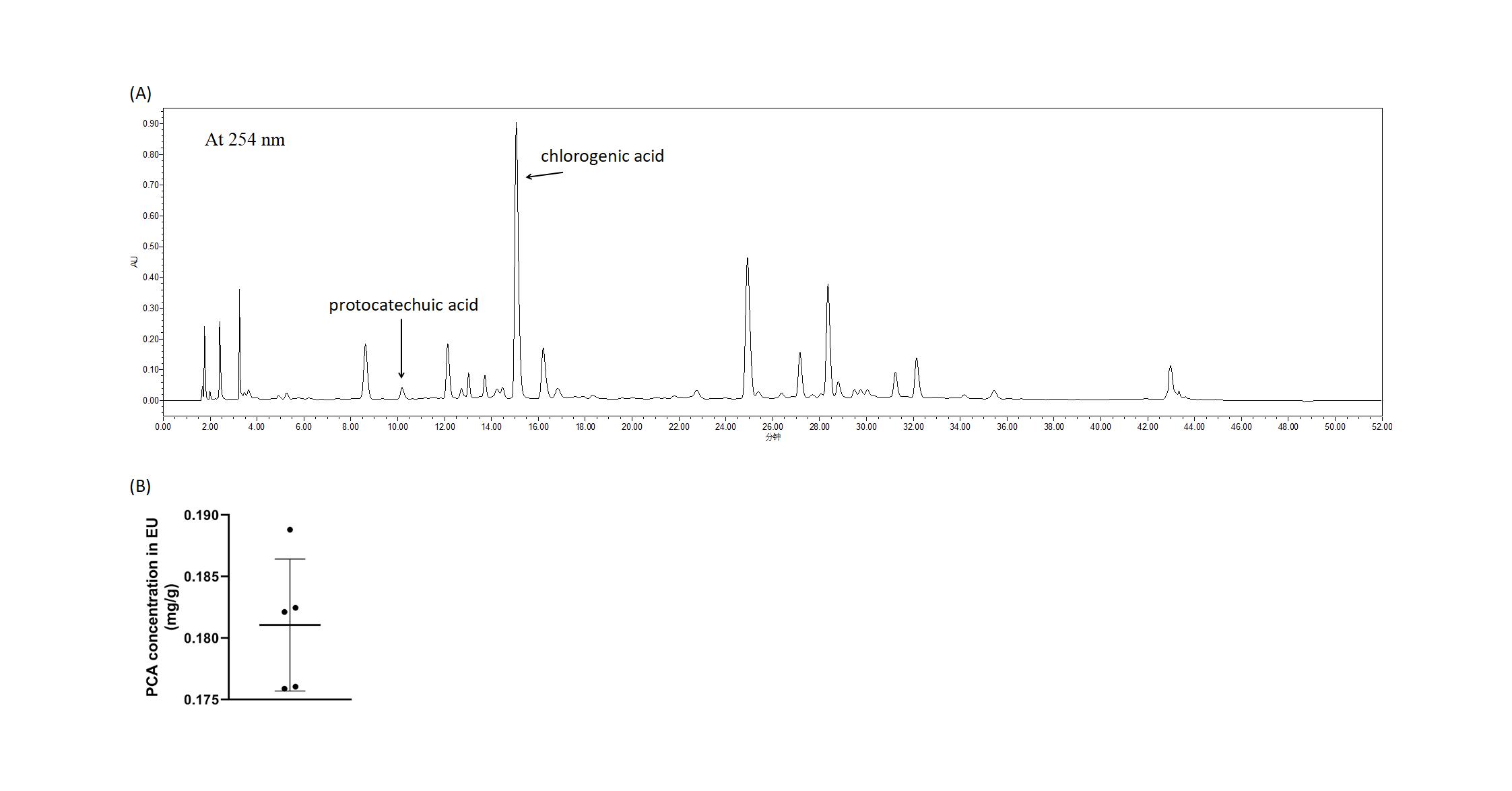

Supplement: Supplementary file 1 [file animals-15-03166-s001.zip › Figure S1.jpg]

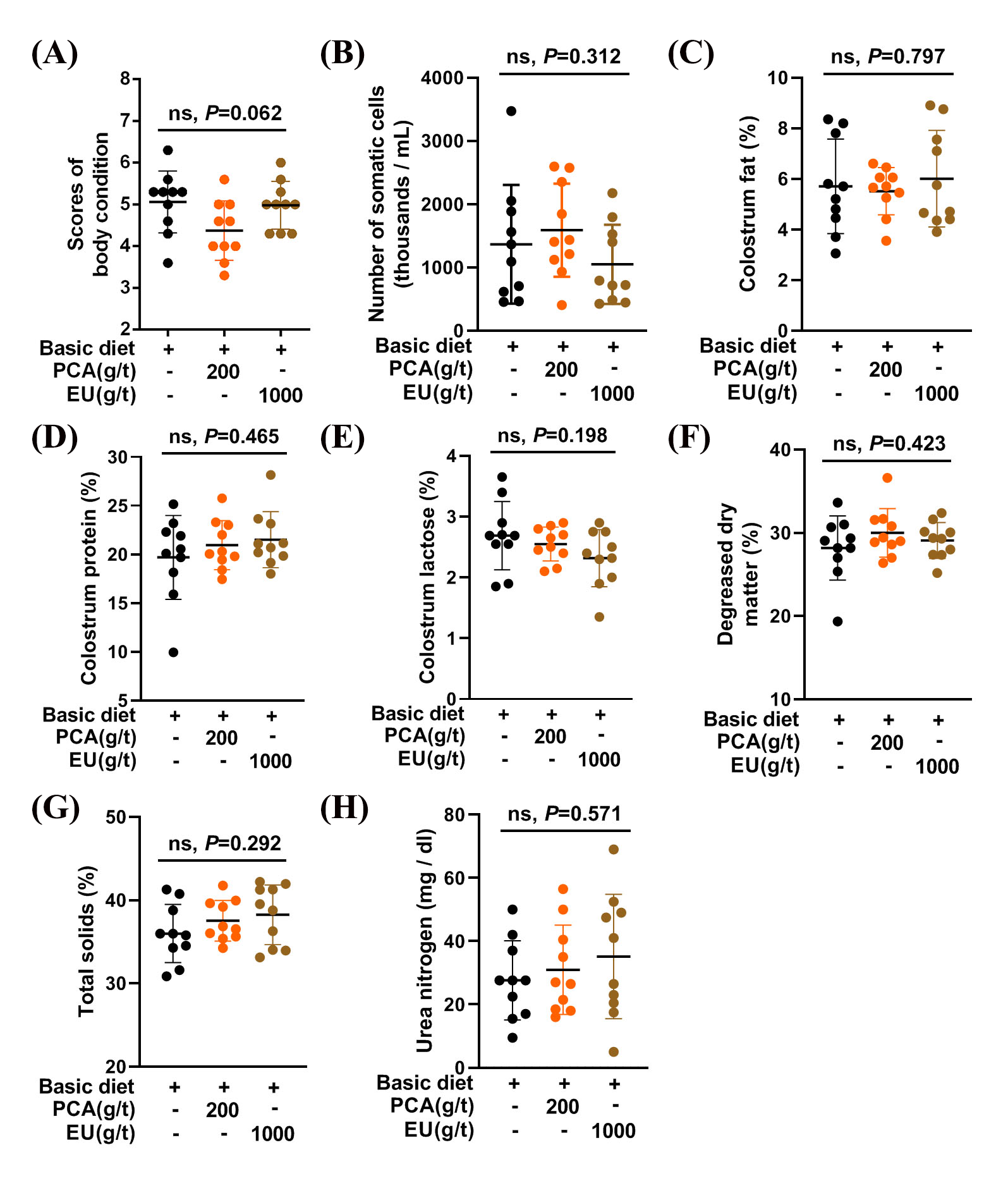

Supplement: Supplementary file 1 [file animals-15-03166-s001.zip › Figure S2.tif]
